# Burden of hepatitis B virus and syphilis co-infections and its impact on HIV treatment outcome in Ethiopia: nationwide community-based study

**DOI:** 10.1080/07853890.2023.2239828

**Published:** 2023-07-27

**Authors:** Yimam Getaneh, Fentabil Getnet, Minilik Demissie Amogne, Lingjie Liao, Feng Yi, Yiming Shao

**Affiliations:** a State Key Laboratory for Diagnosis and Treatment of Infectious Diseases, National Clinical Research Center for Infectious Diseases, Collaborative Innovation Center for Diagnosis and Treatment of Infectious Diseases, The First Affiliated Hospital, College of Medicine, Zhejiang University, Hangzhou, China; b Ethiopian Public Health Institute, Addis Ababa, Ethiopia; c Takemi Program in International Health, Harvard T.H. Chan School of Public Health, Boston, MA, USA; d Lund University, School of Public Health, Lund, Sweden; e State Key Laboratory for Infectious Disease Prevention and Control, National Center for AIDS/STD Control and Prevention, Chinese Center for Disease Control and Prevention, Beijing, China

**Keywords:** Co-infection, HBV, HIV/AIDS, syphilis, Ethiopia

## Abstract

**Background:**

Hepatitis B virus (HBV) and syphilis have been the most common co-infections that hinder treatment outcomes and increase early mortality among people living with human immunodeficiency virus (PLHIV). In this study, we aimed to determine the burden of HBV and syphilis co-infections and its impact on treatment outcomes among PLHIV in Ethiopia.

**Methods:**

We used data from the Ethiopian Population-based HIV Impact Assessment (EPHIA), which was a household-based national survey in 2017/2018. Human immunodeficiency virus (HIV) testing was done among 19,136 participants using the national testing algorithm and 662 participants (3.50%) were HIV positives who were further tested for viral hepatitis and syphilis co-infections using HBV surface antigen and Chembio DPP syphilis assay, respectively. Viral load, CD4 count and high-sensitivity C-reactive protein (hsCRP) were done to measure HIV treatment outcomes. Descriptive statistics were used to determine the burden of co-infections and a logistic regression model to evaluate the determinants of co-infections using STATA V17.0.

**Results:**

Overall prevalence of HBV and syphilis co-infection was 5.5% and 2.2%, respectively. HBV and syphilis (double co-infection) was 5.9%. The highest prevalence of HBV co-infection was observed among 10–19 years age group (12.9%) and male participants (7.44%) while the highest syphilis co-infection was among people aged ≥50 years (3.5%) followed by age groups 40–49 (3.3%) and 10–19 years (3.2%). Syphilis co-infection was higher among males (5.2%) compared to females (1.1%). After adjusted regression analysis, HBV co-infected PLHIV had higher odds of virologic failure (AOR (95% confidence interval (CI)) = 6.3 (4.2–14.3)), immunosuppression (CD4 count < 500 cells/mm^3^) (AOR (95%CI) = 2.1(1.3–4.9)) and inflammation (hsCRP >10 mg/dL) (AOR (95%CI) = 9.2(4.3–14.6)). Immunosuppression was also significantly higher among syphilis co-infected PLHIV (AOR (95%CI) = 3.4 (1.3–5.2)).

**Conclusions:**

Burden of HBV and syphilis co-infections is high particularly among male and adolescent PLHIV and these co-infections hinder virologic and immunologic outcome in Ethiopia. Hence, the program shall enhance HBV and syphilis testing and treatment.

## Background

Human immunodeficiency virus (HIV), hepatitis B virus (HBV) and syphilis are priority global health problems [1], which are commonly called sexually transmitted infections (STIs) [2]. Globally, an estimated 38.4 million people were living with HIV (PLHIV) in 2021. An estimated 0.7% of adults aged 15–49 years worldwide are living with HIV although the burden considerably varies between countries and regions [3]. Hepatitis B virus infects about 296 million people globally [4] while syphilis caused over 14 million illnesses in 2019 an approximately 60% increase from 8.8 million in 1990 [5].

Studies have demonstrated a relationship between HIV and a number of STIs, including HBV and syphilis [1,2,6–8]. The prevalence of HBV and syphilis co-infections was reported to be 10% and 13% in sub-Saharan Africa (SSA), respectively [9,10]. Studies in Ethiopia showed HIV and HBV co-infection is common among children, pregnant mothers and blood donors [1,6,11–15]. Studies also reported the high burden of HBV and syphilis co-infection among elderly (≥50 years old) [9,10,16,17].

If not early diagnosed and treated, HBV and syphilis co-infections can cause severe complications, leading to high rates of morbidity and mortality [9]. HBV and syphilis co-infections have potential risks to increase plasma HIV-1 RNA levels [16]. Studies among PLHIV taking highly active antiretroviral treatment (HAART) indicated that HBV and syphilis co-infections could impact treatment outcomes and increase disease progression and fuel mortality among PLHIV [7–9].

Previous studies were conducted among specific group of population including blood donors [15], children [17] and pregnant women [13,14]. Moreover, they did not address the impact of these co-infections on treatment outcome. Risk factors for the co-infections were also not thoroughly investigated. Therefore, the aim of the current study was to determine the prevalence of HBV and syphilis co-infections among PLHIV and evaluate their impact on HIV treatment outcome at community level.

## Methods

### Study design

**Figure 1.**
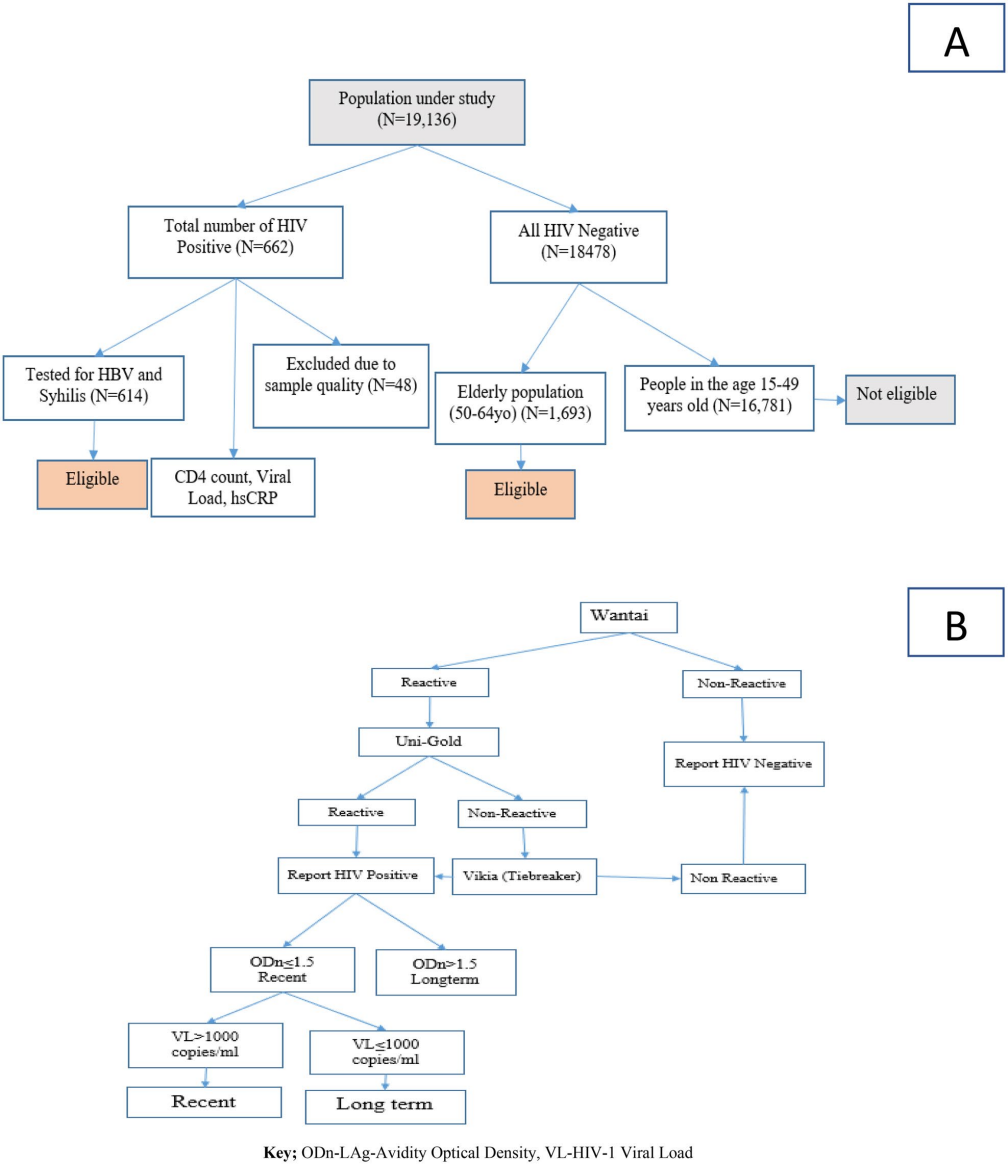
(A) Sampling and sample size determination and (B) detection of recent and long-term infection-HIV-1 LAg Avidity plus VL algorithm.

In 2017/2018, Ethiopia conducted the Ethiopian Population-based HIV Impact Assessment (EPHIA), which was a nationwide household (HH) survey among urban dwellers. In the EPHIA study, HBV and syphilis testing were done for people diagnosed positive for HIV. CD4 count, viral load and high-sensitivity C-reactive protein (hsCRP) tests were also done to measure treatment outcome among PLHIV [18,19] (Figure 1(A)).

Criteria for inclusion of subjects 15–64 years: resides in selected HH or spent the night there the night before the survey, per the above definitions, and self-reported age 15–64 years, and for those between 18 and 64, is able and willing to provide written informed consent. For adolescents 15–17 years old, able and willing to provide written assent and parent/guardian able and willing to provide written informed consent/permission.

### Sampling and sample size determination

EPHIA used a two-stage, age-stratified and cluster sampling approach. The sampling frame accounted all HHs in urban areas based on the 2007 Population and Housing Census in Ethiopia. The study considered 17,339 enumeration areas (EAs) consisting of 3,025,379 HHs and 11,862,821 individuals (i.e. an average of 175 HHs and 684 individuals per EA).

The first stage selected 395 EAs using a probability proportional to population size of the regions, including nine administrative regions (Tigray, Afar, Amhara, Oromia, Ethiopian Somali, Benishangul-Gumuz, SNNPR, Gambella and Harari) and two city administrations (Addis Ababa, and Dire Dawa). In the second stage, a sample of HHs was randomly selected from each EA using an equal probability method, 30 HH per EA and the actual number per EA ranging from 15 to 60.

The sample size was calculated to provide a national estimate of viral load suppression (VLS) with assumed prevalence of VLS = 50% at 95%CI of ±5%. Accordingly, the target sample size was 18,139 for adults (15–49 years old) and 1777 for elderly (50–64 years old). We finally collected data from 19,136 participants with response rate of 96.1% [20]. Details about the sampling sample size determination were published (EPHIA_Report_280820_Web.pdf (columbia.edu)). Among those, 662 study participants were HIV positive who were part of this analysis to determine prevalence of HBV and syphilis co-infections (Figure 1(A)).

### Data collection

Field laboratory and questionnaire data were collected on mobile tablet devices using a programmed application in Open Data Kit (ODK). Interview was administered to people aged ≥15 years old that included demographic characteristics, sexual and reproductive health, marriage, male circumcision, sexual activity, HIV/acquired immune deficiency syndrome (AIDS) knowledge and attitudes, the HIV testing and treatment history. Adolescent questionnaire (for children aged 12–14 years old), contained questions from the adult questionnaire, which included sexual activity, demographic characteristics, exposure to HIV prevention programs and HIV-related risk behaviours. The questionnaire was administered in the most common five languages used in Ethiopia (Amharic, Oromiffa, Tigrigna, Afarigna and Somaligna).

### Laboratory testing

#### Blood sample collection, transport and storage

Whole blood was collected in two ethylenediaminetetraacetic acid (EDTA)-coated test tubes of 3–5 mL each, and plasma was extracted using centrifugation at 2000 revolutions per minute (RPM). Two separate aliquots of the plasma were taken and, one was sent to the regional site for testing while the other to the national reference laboratory for storage at −80 °C. The samples were labelled and stored in temperature-controlled boxes at the field level, transported to a testing laboratory.

### HIV testing

HIV testing and counselling (HTC) was conducted in the selected HHs using the Ethiopian national HIV testing algorithm for rapid-testing: Wantai (Beijing Wantai Biological Pharmacy Enterprise Co., Ltd., Beijing, China) as the first test for screening, then Uni-Gold (HIV 1/2™, Trinity Biotech Plc., Wicklow, Ireland) as confirmatory assay. Finally, Vikia (HIV 1/2, bioMérieux, SA, Marcy-l’Etoile, France) was used as a tiebreaker (Figure 1(B)).

### CD4 count and viral load testing

CD4 count measurement was done for PLHIV in the field using CD4 Analyzer (Pima™, Abbott Molecular Inc., Chicago, IL, formerly Alere). HIV-1 VL (HIV RNA copies per mL) was measured from the plasma using Roche (COBAS^®^ AmpliPrep/COBAS^®^ TaqMan^®^ HIV-1 Test, Roche Diagnostics, Indianapolis, IN).

### ARV detection

For the purpose of classifying recent and long-term infections and identifying whether people are taking HAART, qualitative screening for antiretrovirals (ARVs) was conducted from dried blood spot (DBS) using high-resolution liquid chromatography, which was coupled with tandem mass spectrometry. Testing for ARVs was performed at the Division of Clinical Pharmacology of the Department of Medicine (University of Cape Town in South Africa).

### HBV and syphilis testing

Blood samples were tested for HBV surface antigen (HBsAg) using a testing device Determine (Alere Medical Co., Ltd., Chiba, Japan), One Step Strip HBsAg test which had a specificity of 99.9% and sensitivity of 99.5% [21]. Moreover, samples were also tested for syphilis using a strip Chembio Dual Path Platform (DPP) Syphilis Assay (Hauppauge, New York, NY) that had a sensitivity 98.8% and specificity 99.4% [22].

### Recency testing

Recent infection was estimated based on the number of PLHIV as recent with the HIV-1 VL algorithm plus LAg Avidity as well as ARV detection in blood. This was a technique approved by The World Health Organization (WHO) and obtained using the formula recommended by the WHO Incidence Working Group [23]. Accordingly, the assay performance characteristic of a mean duration of recent infection was (MDRI = 130 days) (Figure 1(B)).

### Statistical analysis

Statistical analysis was performed using STATA V17.0 (StataCorp, College Station, TX). Descriptive statistics were used to provide summary measures (frequencies, means, median and IQR). Multivariable logistic regression model was conducted to evaluate determinants of HBV and syphilis co-infection and assess their impact on HIV treatment outcome. Predictor variables with a significant level <0.2 in bivariable analysis were considered for inclusion of the multivariable regression (i.e. adjusted odds ratio). Each of the outcome variables was adjusted for each and statistical significance was determined at *p* value of <.05.

Predetermined cutoff values were used to characterize viral load, CD4 count and hsCRP. Briefly, virologic failure was defined if viral load >1000 copies/mL, immunosuppression if CD4 count of <500 cells/mm^3^ [24,25], and disease progression or inflammation if hsCRP >10.00 mg/dL [26–28]. We further classified the CD4 count, viral load and hsCRP in to different ranges to identify specific ranges and link with the different co-infections to further characterize treatment outcome.

## Results

### Demographic characteristics

A total of 19,136 study participants included in the EPHIA study, of whom about two-third of the study participants were female (Table 1). Of the total, 662 were HIV positives, means the proportion of HIV positives was 3.5% (95%CI = 3.2–3.7%) among sampled urban dwellers in Ethiopia with significant difference in between male (1.9%) and female (4.1%) participants (Table 2). All HIV positives were eligible for HBV and syphilis testing, but 48 had sample quality problems, so 614 HIV positives were finally included in the co-infection analysis. Among the HIV positive population, a quarter of the study participants were from Oromia (24.3%) region followed by Amhara (19%) and Addis Ababa (14%).

**Table 1.** Demographic characteristics among people living with HIV in Ethiopia (2017/2018).

		Frequency	Percent
Region	Tigray	39	6.4
	Afar	32	5.2
	Amhara	118	19.2
	Oromia	149	24.3
	Somali	8	1.3
	Benishangul-Gumuz	20	3.3
	SNNPR	49	8.0
	Gambella	44	7.2
	Harari	32	5.2
	Addis Ababa	88	14.3
	Dire Dawa	35	5.7
Sex	Female	461	75.1
	Male	153	24.9
Age	10–19	31	5.0
	20–29	105	17.1
	30–39	238	38.8
	40–49	154	25.1
	≥50	86	14.0
Marital status	Never married	71	11.6
	Married or living together	285	46.6
	Divorced or separated	144	23.5
	Widowed	112	18.3
Education level	No education	121	19.8
	Primary	291	47.6
	Secondary	141	23.1
	More than secondary	58	9.5
Wealth quantile	Lowest	103	16.8
	Second	108	17.6
	Middle	143	23.3
	Fourth	147	23.9
	Highest	113	18.4
Number of sexual partners in 12 months	0	241	45.0
	1	270	50.4
	≥2	25	4.7
Condom use in 12 months	Used condom	83	15.7
	Did not use condom	203	38.5
	No sex in the past 12 months	241	45.7
Relationship status	Not spouse/live-in partner	60	20.3
	Spouse/live-in partner	235	79.7
Circumcision status	Yes	143	23.3
	No	8	1.3
Diagnosed with STI	Yes	28	4.6
	No	549	89.4
Viral suppression	Not viral load suppressed	182	29.6
	Viral load suppressed	432	70.4
Recent infection	Long term	607	98.9
	Recent	7	1.1
CD4 < 350	Yes	221	36.0
	No	393	64.0
CD4 < 500	Yes	377	61.4
	No	237	38.6
Know HIV status	Un aware	147	25.4
	Aware	431	74.6
On HAART	Not on ARVs	168	29.1
	On ART	410	70.9
hsCRP	≤10.00	483	78.7
	>10	131	21.3
	Total	614	100.0

**Table 2.** Burden of HIV, HBV and syphilis co-infection among PLHIV in Ethiopia (2017/2018).

Characteristics		Positive	Negative	HIV				HBV			Syphilis		
				Total	Positive (%)	Positive	Negative	Total	Positive (%)	Positive	Negative	Total	Positive (%)
Sex	Female	476	1123	11,599	4.1	22	439	461	4.77	456	5	461	1.08
	Male	143	7394	7537	1.9	12	141	153	7.84	145	8	153	5.23
Age (years)	10–19	69	945	1014	6.80	4	27	31	12.90	30	1	31	3.23
	20–29	55	7492	7547	0.73	6	99	105	5.71	103	2	105	1.90
	30–39	495	16,731	17,226	2.88	14	224	238	5.88	236	2	238	0.84
	40–49	84	1826	1910	4.41	8	146	154	5.19	149	5	154	3.25
	≥50	579	18,557	19,136	4.40	2	84	86	2.33	83	3	86	3.49
Marital status	Never married	69	7034	7103	0.97	6	65	71	8.45	69	2	71	2.82
	Married or living together	234	8248	8482	2.76	13	272	285	4.56	278	7	285	2.46
	Divorced or separated	30	906	936	3.18	7	137	144	4.86	142	2	144	1.39
	Widowed	134	1589	1723	7.75	8	104	112	7.14	110	2	112	1.79
Education level	No education	124	2276	2400	5.16	7	114	121	5.79	119	2	121	1.65
	Primary	287	6516	6803	4.22	11	280	291	3.78	285	6	291	2.06
	Secondary	131	5357	5488	2.38	13	128	141	9.22	139	2	141	1.42
	More than secondary	43	4333	4376	0.99	3	55	58	5.17	55	3	58	5.17
Wealth quintile	Lowest	104	3224	3328	3.13	8	95	103	7.77	100	3	103	2.91
	Second	107	3322	3429	3.13	3	105	108	2.78	107	1	108	0.93
	Middle	135	3675	3810	3.53	7	136	143	4.90	141	2	143	1.40
	Fourth	135	4014	4149	3.25	10	137	147	6.80	146	1	147	0.68
	Highest	101	4319	4420	2.27	6	107	113	5.31	107	6	113	5.31
Number of sexual partners in the past 12 months	0	221	3468	3689	6.0	14	227	241	5.81	237	4	241	1.66
	1	255	8523	8778	2.9	14	256	270	5.19	264	6	270	2.22
	≥2	16	481	497	3.1	0	25	25	0.00	24	1	25	4.00
Viral load suppression	Not viral load suppressed					15	167	182	8.24	176	6	182	3.30
	Viral load suppressed					19	413	432	4.40	425	7	432	1.62
Recent/long term infection	Long term					32	575	607	5.27	594	13	607	2.14
	Recent					2	5	7	28.57	7	0	7	0.00
CD4 < 350	Yes					13	208	221	5.88	216	5	221	2.26
	No					21	372	393	5.34	385	8	393	2.04
CD4 < 500	Yes					19	358	377	5.04	366	11	377	2.92
	No					15	222	237	6.33	235	2	237	0.84
On HAART	Not on ARVs					9	159	168	5.36	161	7	168	4.17
	On ART					22	388	410	5.37	405	5	410	1.22
hsCRP	≤10.00					11	472	483	2.28	32	2	34	5.88
	>11.00					23	108	131	17.56	569	11	580	1.90
Total		664	18,557	19,136	3.02	34	580	614	5.54	601	13	614	2.12

### HBV and syphilis co-infection among HIV positive people

**Figure 2.**
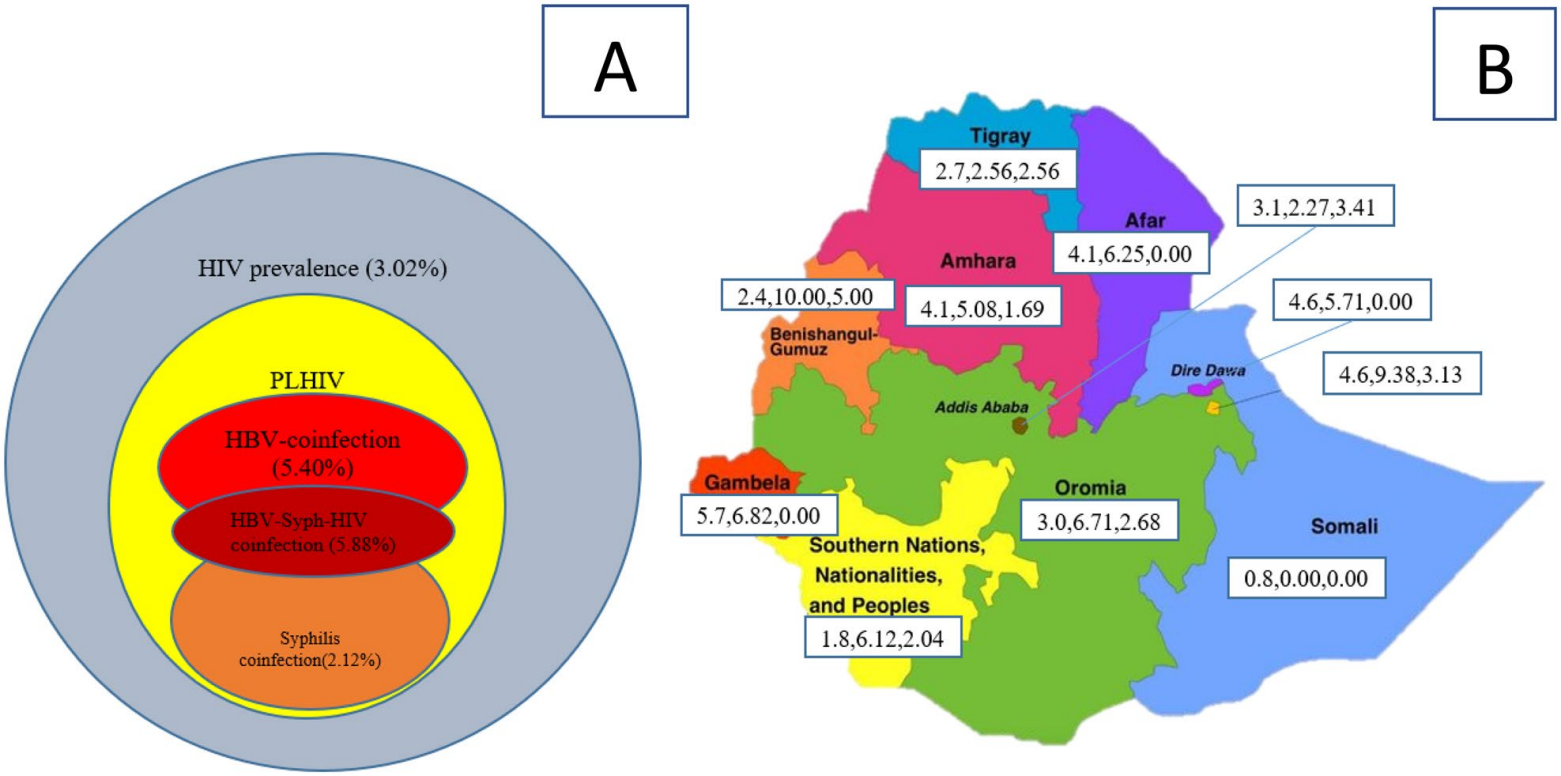
(A) Overall prevalence of HIV, HBV and syphilis co-infection in urban Ethiopia and (B) HIV, HBV and syphilis co-infection disaggregated by region.

Overall prevalence of HBV among PLHIV was 5.5%. The co-infection was 7.8% among males and 4.8% among females. The highest prevalence of HBV co-infection was observed among the age group 10–19 years (12.9%). HBV co-infection was also higher among never married (8.8%) and divorced/widowed (7.1%). The lowest wealth quintile had the highest HBV co-infection (7.8%). Double-HBV-syphilis co-infection was 5.9% (Table 2, Figure 2(A)).

The overall prevalence of syphilis co-infection was 2.2%, it was higher among male (5.2%) compared to 1.1% among female. The co-infection was higher among people aged ≥50 years (3.5%), with multiple sexual partners (4.0%), with no viral suppression (3.3%), with CD4 count <500 cells/mm^3^ (2.9%) and those who were HAART-naive (4.2%) (Table 2, Figure 2(A)).

### HIV, HBV and syphilis co-infection disaggregated by region

HBV and syphilis co-infections were heterogeneous among the different regional administrations in the country. The highest prevalence of HBV co-infection was observed in Benishangul-Gumuz, Harari and Oromia regions with 10.0%, 9.4% and 6.7%, respectively. The highest prevalence of syphilis co-infection was also in Benishangul-Gumuz, Addis Ababa and Harari with 5.0%, 3.4% and 3.1%, respectively (Figure 2(B)).

### Risk factors of HBV and syphilis co-infection

The odds of HBV co-infection among HIV positives were higher among males (AOR (95%CI) = 2.7 (1.7–4.0)), adolescents (AOR (95%CI) = 5.22 (2.03–11.43)) and syphilis positives (AOR (95%CI) = 2.4 (1.1–6.8)). The odds of syphilis co-infection were also higher in those with CD4 count <500 cells/mm^3^ (AOR (95%CI) = 2.5 (1.5–11.7)) and HAART-naive (AOR (95%CI = 3.1 (1.2–10.2) (Table 3).

**Table 3.** Predictors of HBV and syphilis co-infections among PLHIV in Ethiopia (2017/2018).

Characteristics		Predictors of HBV-HIV co-infection								Predictors of syphilis-HIV co-infection							
		95%CI				95%CI				95%CI				95%CI			
		Sig.	COR	Lower	Upper	Sig.	AOR	Lower	Upper	Sig.	COR	Lower	Upper	Sig.	AOR	Lower	Upper
Gender	Female	Ref.															
	Male	0.06	0.41	0.16	1.04	0.03	2.69	1.71	3.99								
Age	10–19	0.05	9.21	0.97	86.85	0.11	0.22	0.03	1.43								
	20–29	0.08	4.74	0.80	27.82	0.71	0.72	0.12	4.08								
	30–39	0.08	3.98	0.81	19.42	0.23	0.39	0.08	1.86								
	40–49	0.19	2.93	0.58	14.81	0.50	0.57	0.11	2.90								
	≥50	Ref.															
Wealth quantile	Lowest	0.93	0.94	0.23	3.80	0.69	1.52	0.18	12.25	0.08	0.14	0.01	1.29	0.69	1.52	0.18	12.25
	Second	0.22	0.37	0.07	1.83	0.14	6.77	0.51	88.28	0.56	0.64	0.14	2.8	0.14	6.77	0.51	88.28
	Middle	0.45	0.60	0.16	2.22	0.14	4.80	0.57	39.90	0.21	0.24	0.02	2.21	0.14	4.80	0.57	39.90
	Fourth	0.85	0.89	0.27	2.89	0.06	8.46	0.85	83.50	0.21	0.34	0.06	1.84	0.06	8.46	0.85	83.50
	Highest	Ref.				Ref.								Ref.			
Viral load suppression	Not viral load suppressed	0.21	2.11	0.65	6.85												
	Viral load suppressed	Ref.															
Recency	Long term	0.09	0.17	0.02	1.34												
	Recent	Ref.															
CD4 < 350	Yes	0.63	1.33	0.40	4.34												
	No	Ref.															
CD4 < 500	Yes	0.06	0.34	0.11	1.07	0.02	2.78	1.14	6.79	0.16	0.29	0.043	1.66	0.01	2.45	1.51	11.71
	No	Ref.								Ref.							
ARV status	Not on ARVs	0.26	0.24	0.02	2.95					0.03	0.11	0.01	0.83	0.03	3.05	1.18	10.17
	On ART	Ref.								Ref.							
Syphilis	Negative	0.06	0.18	0.02	1.14	0.03	2.42	1.35	6.43								
	Positive	Ref.															
hsCRP	≤10.00	0.00	0.10	0.04	0.23	0.00	10.08	4.50	22.60								
	≥11.00																
HBV status	Positive									0.04	0.14	0.02	0.95	0.01	4.08	1.79	21.01
	Negative									Ref.							

CI: confidence interval; COR: crude odds ratio; AOR: adjusted odds ratio; hsCRP: high-sensitivity C-reactive protein; ARV: antiretroviral.

### Impact of HBV and syphilis co-infection on HIV treatment outcome

Higher rates of HIV virologic failure (44.1%), immune suppression 44.3% and inflammation (67.6%) were observed among HBV co-infected people compared to the respective values of 28.8%, 38.3% and 18.6% among HBV negatives (Table 4, Figure 2(B)). In other words, being HBV coinfected was associated with high virologic failure (AOR (95%CI) = 6.3 (4.2–14.3)), risk immunosuppression (AOR (95%CI) = 2.1 (1.3–4.9)) and occurrence of inflammation (AOR (95%CI) = 9.2 (4.3–14.6)).

**Figure 3.**
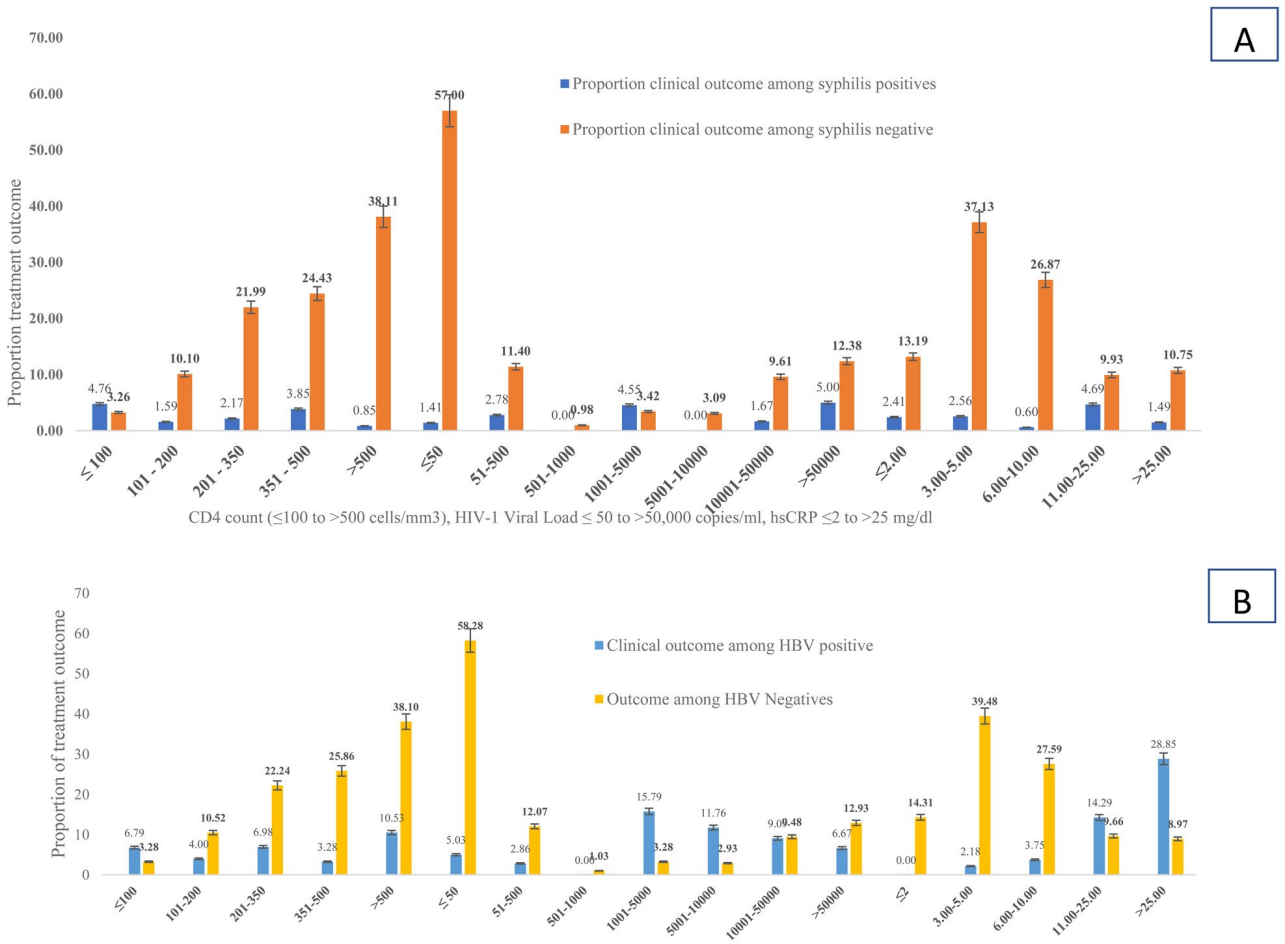
Treatment outcome disaggregated by HBV and syphilis status among PLHIV in Ethiopia. (A) Treatment outcome disaggregated by HBV status and (B) treatment outcome disaggregated by syphilis status.

The rates of HIV virologic failure (46.2%), immunosuppression (39.1%) and inflammation (30.8%) were also significantly higher among syphilis positive patients compared to their respective values of 29.3%, 15.4% and 21.13% among the syphilis negatives (Table 4, Figure 3(B)).

**Table 4.** Impact of co-infections on treatment outcome among PLHIV in Ethiopia (2017/2018).

Infection		Viral load suppression (copies/mL)							CD4 count (cells/mm^3^)					hsCRP (mg/dL)						
		Not viral load suppressed		Viral load suppressed		Total	Virologic failure (%)	OR (95%CI)	CD4 ≥ 500	CD4 < 500	Total	CD4 < 500 (%)	OR (sig.)	≤10.0	>11.0		Total		High CRP (%)	OR (sig.)
Active syphilis	Negative	176	425		601		29.28	Ref.	366	235	601	15.38	Ref.	474		127		601	21.13	Ref.
	Positive	6	7		13		46.15	2.21 (0.61, 4.28)	11	2	13	39.10	3.38 (1.26, 5.22)	9		4		13	30.77	0.64 (0.21, 2.26)
HBV	Positive	15	19		34		44.12	6.26 (4.21, 14.28)	19	15	34	44.28	2.11 (1.32, 4.86)	11		23		34	67.65	9.21 (4.26, 14.62)
	Negative	167	413		580		28.79	Ref.	358	222	580	38.28	Ref.	472		108		580	18.62	Ref.
Total		182	432		614		29.64		377	237	614	38.60		483		131		614	21.34	

## Discussion

In this study, we have documented the burden of the two sexually transmitted co-infections (i.e. HBV and syphilis) and evaluated their impact on HIV treatment outcome. The co-infection rate of HBV and syphilis was 5.5% and 2.2% among people living with HIV in Ethiopia with significant variation in prevalence across regional states. The odds of HBV co-infection were higher among males, adolescents and syphilis positives, while syphilis co-infection was higher in patients with CD4 count <500 cells/mm^3^ and those who did not initiate HAART, *p* < .05. Both HBV and syphilis co-infections were significantly associated with increased virologic failure, immune suppression and inflammation, *p* < .05.

The overall prevalence of HBV co-infection in the current study (5.5%) was concordant with findings in various localities of Ethiopia: Addis Ababa (3.9%) [29], Mekelle (5.9%) [21] and Gondar (5.6%) [30]. The similar prevalence of HBV among the general population (7%) [31] or other population groups such as pregnant women (3.8–6%) [11] and blood donors (4.7–10.9%) [32] highlights that HIV infected people might not be different from other population segments with respect to the burden of HBV. Moreover, the current prevalence of HBV is lower compared to the 15% among HIV-infected people in sub-Sahara Africa, which might be due to the epidemiologic differences across countries [2], and the difference might also be affected by difference in the HAART contents and effectiveness across country because certain HIV drugs such as lamivudine can reduce HBV incidence. Nonetheless, the observed magnitude of HBV is of concern and requires interventions to reduce consequences since the absolute number of people living with HIV in Ethiopia is large (*N* = 609,349) [33].

The regional disparities in HBV co-infections that ranged from 0.0% in Somali to 10.0% in Benishangul-Gumuz was consistent with the prevalence of HIV among the general population. This could be explained by similarity with the route of transmission of HIV and HBV [34]. On the hand, this study identified a relatively higher burden of HBV co-infection among male (7.8%) compared to women (4.8%) which is consistent with a previous study [35]. However, this was contrary to HIV prevalence in Ethiopia, which is higher in female (4.1%) than male (1.9%) [36]. This may require further investigation to answer why HBV co-infection is higher in male unlike HIV mono-infection. Furthermore, the highest prevalence of HBV co-infection among adolescents aged 10–19 years (12.90%) highlights the needs to strengthening targeted HBV vaccination and enhancing school-based interventions.

The 2.2% syphilis co-infection in our study is similar to the 2.3% national prevalence among the general population in Ethiopia [13]. Other studies done in different areas of Ethiopia have also shown similar prevalence among pregnant women (2.3%) [29], 2.9% [13] and 3.9% [21]. The prevalence of syphilis co-infection was the highest among people in the aged ≥50 years (3.5%) followed by age groups of 40–49 (3.3%) and 10–19 years (3.2%). Moreover, the higher syphilis co-infection among adolescents might be explained by recent high HIV infections, which highlight the interplay between new HIV infection with syphilis co-infections [37].

The higher risk HBV co-infection among male (AOR (95%CI) = 2.7 (1.7–4.0)) is supported by similar studies in Zimbabwe [35] and South Africa [38]. This could be due to the fact that vaccination program for HBV was not inclusive of men. Moreover, CD4 count <500 cells/mm^3^ was also a risk for acquiring active syphilis (AOR = 2.45, 95%CI = 1.51, 11.71), which could be explained by syphilis reactivation as immunosuppression [1]. On the other hand, syphilis infection itself was a risk for acquiring HBV (AOR (95%CI) = 2.4 (1.1–6.8)), which is also consistent with previous studies [1,18]. The genital ulcers associated with syphilis infection might facilitate the transmission of HBV infection [16].

Co-infection of HBV with HIV complicates the clinical course, management and also adversely affect therapy for HIV infection [30,31]. Virologic failure was substantially higher among HBV co-infected people (44.1%) compared to the 28.8% among HBV negatives. Similarly, the 46.2% virologic failure among syphilis co-infected patients was substantially higher (AOR (95%CI) = 6.3 (4.2–14.3)) compared to the 29.3% in syphilis non-infected. This was consistent with a previous systematic review [39]. This emphasizes the need for HBV and syphilis screening as part of the clinical management of HIV positives to improve virologic outcomes.

Similarly, immune suppression was higher among both HBV coinfected (AOR (95%CI) = 2.1 (1.3–4.9)), and this is consistent with previous findings [2,16,31]. Moreover, it was also higher and syphilis co-infected patients (AOR (95%CI) = 3.4 (1.3–5.2)), and it is supported by reports in SSA [40,42]. Beyond the impact on treatment outcome, this study highlighted the effect of immune suppression on syphilis reactivation which was also previously reported [41].

In addition, HBV positives had higher levels of hsCRP (AOR (95%CI) = 9.2 (4.3–14.6)). This may indicate the impact of HBV co-infection on the high level of inflammation which could lead to the different types of toxicities as revealed by different studies [28,45,46].

This was the first community based nationwide study that could provide better insight about the burden of HBV and syphilis co-infections and its impact on HIV treatment outcome to the program and scientific community. However, since prevalence of HIV in the country was concentrated in urban Ethiopia (i.e. 3.0%) while it was 0.62% in rural, this study was conducted in urban area.

## Conclusions

The rates of HBV and syphilis co-infections among HIV positive are comparable to their prevalence among the general population in Ethiopia. However, our findings highlight that the burden of these co-infections is substantially higher in male and adolescents. This calls for specific program interventions to address most affected population segments such as targeted vaccination for highly affected male and adolescents. HBV and syphilis co-infections hinder HIV virologic and immunologic outcomes. Moreover, immunosuppression and virologic failure in HIV patients can also lead to syphilis reactivation—a threat for high surge of syphilis in HIV positives with poor treatment compliance and responses. Hence, the national HIV/AIDS program shall enhance HBV and syphilis testing for PLHIV as part of the standard clinical management during the follow-up.

## Data Availability

Since data analysis for other objectives is ongoing, the raw data can be obtained from the first corresponding author.
